# Expression Screening of Fusion Partners from an *E. coli* Genome for Soluble Expression of Recombinant Proteins in a Cell-Free Protein Synthesis System

**DOI:** 10.1371/journal.pone.0026875

**Published:** 2011-11-02

**Authors:** Jin-Ho Ahn, Jung-Won Keum, Dong-Myung Kim

**Affiliations:** 1 Department of Fine Chemical Engineering and Applied Chemistry, Chungnam National University, Daejeon, Republic of Korea; 2 School of Chemical and Biological Engineering, Seoul National University, Seoul, Republic of Korea; University of Crete, Greece

## Abstract

While access to soluble recombinant proteins is essential for a number of proteome studies, preparation of purified functional proteins is often limited by the protein solubility. In this study, potent solubility-enhancing fusion partners were screened from the repertoire of endogenous *E. coli* proteins. Based on the presumed correlation between the intracellular abundance and folding efficiency of proteins, PCR-amplified ORFs of a series of highly abundant *E. coli* proteins were fused with aggregation-prone heterologous proteins and then directly expressed for quantitative estimation of the expression efficiency of soluble translation products. Through two-step screening procedures involving the expression of 552 fusion constructs targeted against a series of cytokine proteins, we were able to discover a number of endogenous *E. coli* proteins that dramatically enhanced the soluble expression of the target proteins. This strategy of cell-free expression screening can be extended to quantitative, global analysis of genomic resources for various purposes.

## Introduction

Rapid progress in sequencing technology is generating enormous amounts of sequence data, making protein expression a major bottleneck in the functional analysis of identified genetic resources [1], [2], [3], [4]. When compared to traditional cell-based gene expression, cell-free protein synthesis offers excellent speed and flexibility for parallel expression of multiple proteins [5], [6], [7]. For instance, Kwon *et al*. were recently able to identify novel transaminases from the genomic sequences of *Rhodobacter sphaeroides* and *Mesorhizobium loti* strains by cloning-independent, cell-free expression analysis of computer-predicted putative tranaminase sequences [8]. In theory, cell-free synthesis enables functional interpretation of protein-coding sequences from any genomes with unsurpassed throughput.

However, similar to the case of cell-based gene expression, cell-free synthesized proteins often fail to acquire a native soluble structure, hampering downstream analysis of the translation products. Combining target proteins with a solubility-enhancing partner is one of the most generic, but effective tactics to promote the solubility of recombinant proteins [9]. Many otherwise highly insoluble proteins have been expressed as soluble fusion proteins with a number of solubility-enhancing fusion partners including glutathione-S-transferase (GST) [10]. maltose binding protein (MBP) [11], [12], thioredoxin (Trx) [13], NusA [14], and SUMO protein [15], However, only a few established fusion partners are currently available; thus, the development of novel fusion partners is necessary to enable proficient expression and analysis of rapidly increasing protein-coding sequences.

The use of fusion partners can influence the translational efficiency of the target genes as well as the solubility of the translation products. Indeed, it is well known [16], [17], [18], [19], [20], [21], [22] that the nature of initial codons next to the start codon has a crucial effect on the expression efficiency of the downstream genes [23]. Therefore, both the overall expression level and relative solubility of the target proteins can be altered upon N-terminal fusion with fusion partners. Since neither the effect of the fusion partner sequences on the translation efficiency or solubility of the translation product can be predicted, selection of optimal fusion partners that allow the maximum expression of soluble target proteins requires exhaustive expression studies of different gene constructs. While combinatorial expression analysis has been a challenging task due to the limited throughput of cell-based gene expression, in this study, we conducted large scale cell-free expression screening of solubility-enhancing fusion partners from highly abundant *E. coli* proteins. Among the more than 1,000 different protein species that exist in *E. coli* cells during normal growth, ribosomal proteins and other protein synthesis-related proteins represent the most abundant protein species [24], [25], [26], [27]. For example, ribosomal proteins account for as much as 34% of the total cellular protein mass and approximately 8% of the total cellular volume of *E. coli* [26], [28], [29]. We speculated that these extremely abundant proteins have more efficient folding pathways than other endogenous proteins to enable tolerance of such a high concentration inside the cells. Based on this assumption, it is expected that these proteins could be used as the fusion partners to enhance the soluble expression of heterologous proteins in a cell-free protein synthesis system derived from *E. coli* extracts. During the initial screening, 88 fusion partner proteins were investigated for their ability to improve the expression level and solubility of three model proteins (human β-defensin 2, human epidermal growth factor, and human erythropoietin). Among the 88 tentative fusion partners examined, 12 *E. coli* proteins were found to be exceptionally effective at improving the expression of model proteins in terms of the expression level and solubility. The fusion partners selected during the primary screening were then applied for soluble expression of 24 cytokines, a class of proteins that are extremely difficult to express in soluble forms in the present cell-free synthesis system derived from *E. coli* extract. Through the expression screening analysis of 288 combinatorial fusion constructs (12 fusion partners against 24 cytokines), we were able to select potent fusion partners that enhanced the soluble expression of target proteins by as much as 29 fold. Although the effect of the examined fusion partners appeared to be protein specific, a number of fusion partners led to particularly dramatic improvements in the expression of soluble proteins.

## Materials and Methods

### Materials

ATP, GTP, UTP, CTP, creatine phosphate, creatine kinase and *E. coli* total tRNA mixture were purchased from Roche Applied Science (Indianapolis, IN). L-[U-^14^C]leucine (11.9 GBq/mmol) was obtained from Amersham Biosciences (Uppsala, Sweden). *E. coli* strain BL21-Star™ (DE3) was obtained from Invitrogen (Carlsbad, CA). Oligonucleotides used in this study were synthesized by Integrated DNA technologies on a 25 nmole scale with standard desalting purification. All other reagents were purchased from Sigma (St. Louis, MO) and used without further purification. The S30 extract was prepared from strain BL21-Star™ (DE3) according to previously described methods [17], [23]. cDNAs of human and murine cytokines were obtained from the Bank for Cytokine Research (Chonbuk, Korea).

### PCR construction of expression templates

**Figure 1:**
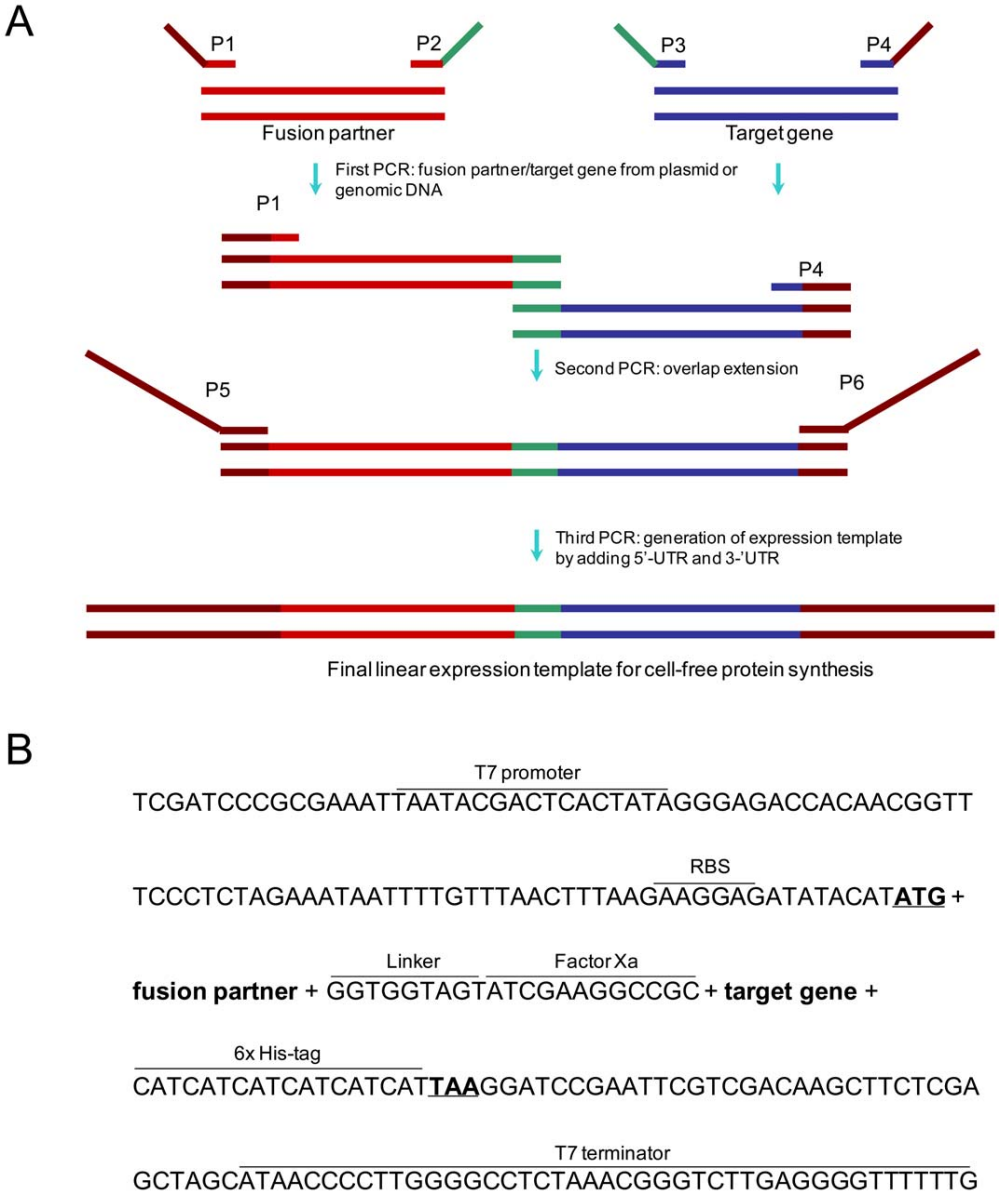
Three-step PCR reactions to assemble linear expression template. (A) Schematic representation of PCR-based generation of fusion constructs. Two primary PCR products with defined overlapping ends are synthesized by the first PCR reaction. These two fragments are joined in a second PCR, overlap extension PCR and subsequent third PCR step introduces the regulatory elements necessary for transcription and translation to the fused target genes. (B) Sequence elements of final amplified expression template.

Combinatorial gene constructs of fusion partners and target proteins were prepared via three-step PCR as shown in Figure 1, after which they were used directly as the expression templates without purification. In the first-round PCR, fusion partner sequences from the genomic DNA of *E. coli* K12 strain and target sequences from the cloned genes were amplified separately. Pairs of fusion partners and model protein genes were then joined and amplified in the subsequent second and third-round PCR reactions, respectively (see Table S1 for the sequences of the primers used in each PCR reaction).

### Cell-free protein synthesis reactions

The standard reaction mixture for cell-free protein synthesis consisted of the following components: 57 mM Hepes–KOH (pH 8.2), 1.2 mM ATP, 0.85 mM each of CTP, GTP, and UTP, 2 mM DTT, 0.17 mg/ml *E. coli* total tRNA mixture (from *E. coli* strain MRE600), 0.64 mM cAMP, 90 mM potassium glutamate, 80 mM ammonium acetate, 12 mM magnesium acetate, 34 µg/ml l–5-formyl-5,6,7,8-tetrahydrofolic acid (folinic acid), 1.0 mM each of 20 amino acids, 2% polyethylene glycol (PEG) 8000, 67 mM creatine phosphate (CP), 3.2 µg/ml creatine kinase (CK), 0.01 mM L-[U-^14^C]leucine (11.9 GBq/mmol, Amersham Biosciences), and 10 µg/ml DNA, 24% (v/v) S30 extract.

The amounts of the cell-free synthesized proteins were determined by measuring the TCA-precipitated radioactivity in 15 µl of reaction sample as previously described [30], [31]. The solubility of the synthesized protein was estimated based on the ratio of the TCA-precipitated radioactivity of the reaction samples before and after centrifugation at 20,000 RCF for 20 min [32].

## Results

### Preparation of combinatorial fusion constructs for cell-free synthesis of aggregation-prone proteins

**Figure 2:**
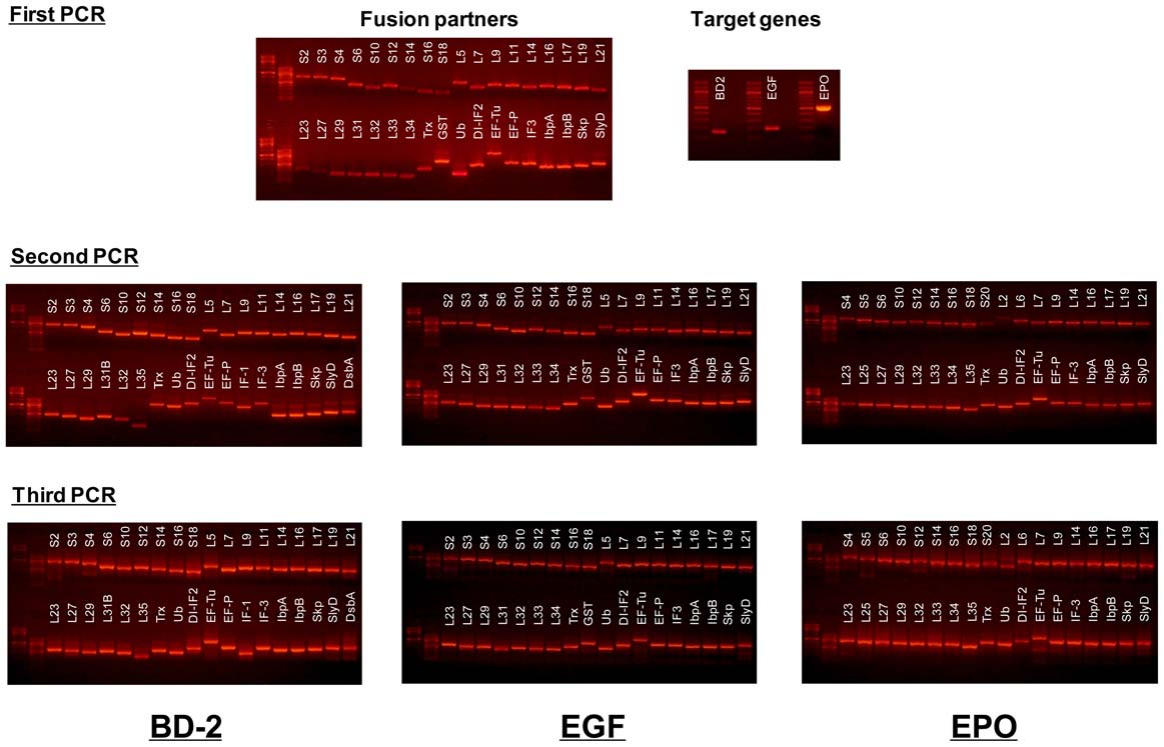
Agarose gel electrophoresis of PCR products.

56 ribosomal proteins, 21 translation-related factors and molecular chaperones, three OB-fold domains, and eight of the most commonly used fusion partner proteins were examined as fusion partners for the expression of aggregation-prone proteins (Table 1). For the initial screening, each of the genes of the tentative fusion partners was fused to the N-termini of three different model proteins, human β-defensin 2 (hBD-2), human epidermal growth factor (hEGF) and human erythropoietin (hEPO). These model proteins were selected because they show very poor expression levels and solubility in the present cell-free protein synthesis system. The DNA constructs used to direct the synthesis of fusion proteins were prepared through three-step PCR procedures using six primers for each fusion construct as outlined in Figure 1A. The constructs were designed to include the T7 promoter and ribosomal binding site in the 5′-UTR and the T7 terminator sequence in the 3′-UTR. In addition, the cleavage site for Factor Xa (ATCGAAGGCCG, Ile-Glu-Gly-Arg) following a short linker (GGTGGTAGT, Gly-Gly-Ser) was introduced between the fusion partner and target protein coding genes (Figure 1B). After being confirmed on an agarose gel for their size and relative amounts (Figure 2), the PCR products coding for each fusion protein were incubated in the reaction mixture for protein synthesis as described in the Materials and Methods.

**Table 1:** Fusion partners used in this study.

Category	Partner protein
Conventional fusion partner (8)	MBP[36], Trx[37], GST[38], NusA[39], Ubiquitin (Ub)[40], Domain I of IF-2 (DI-IF2, 1–158)[41], N-terminal domain of L9 (NTL9, 1–56) [42]
Ribosomal protein (56)	30S ribosomal subunitS1, S2, S3, S4, S5, S6, S7, S8, S9, S10, S11, S12, S13, S14, S15, S16, S17, S18, S19, S20, S21, S2250S ribosomal subunitL1, L2, L3, L4, L5, L6, L7, L9, L10, L11, L13, L14, L15, L16, L17, L18, L19, L20, L21, L22, L23, L24, L25, L27, L28, L29, L30, L31, L31B, L32, L33, L34, L35, L36
Translation-related factor (4)	EF-Tu, EF-P, IF1, IF3
Chaperone protein (17)	IbpA, IbpB, Skp, SlyD, DsbA, DsbB, DsbC, SecB, SecE, SecG, GrpE, FkpB, FklB, GroEL, GroES, GroEL191–345, GroEL191–376
OB-fold domain (3)*	LysN1–145, AspN1–102, AsnN1–99

*This family contains OB-fold domains that bind to nucleic acids (oligonucleotide/oligosaccharide-binding fold). The family includes the anti-codon binding domain of lysyl, aspartyl, and asparaginyl-tRNA synthetases [43].

### Effect of fusion partners on the solubility and expression level of target proteins

**Figure 3:**
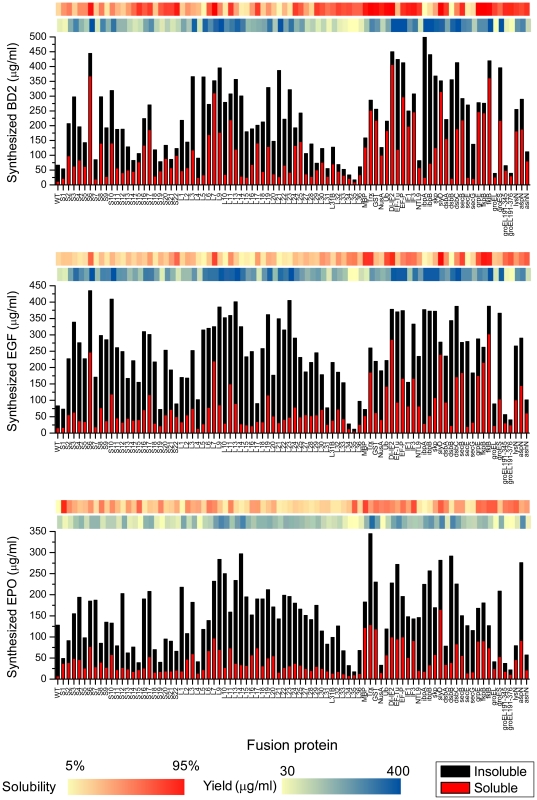
Solubility and expression efficiency of fused gene constructs. Eighty seven fusion partner genes were combinatorially fused to three different genes (hBD2, hEGF, hEPO) using three-step PCR. All PCR products coding each fused gene were directly used as expression templates for cell-free protein synthesis where expression efficiency and solubility were measured. After 3 h for cell-free expression, the reaction samples were centrifuged at 10,000 rpm for 30 min. Both pellet and soluble fractions were analyzed by radioactivity counting. The degree of solubility and expression yield enhancement for each fusion gene is colorized with red and blue respectively.

As shown in Figure 3, both the expression level and solubility of the target proteins showed drastic variations in the presence of different fusion partners (Tables S2, S3, S4). For example, in the case of hBD-2 expression, the greatest increase in the amount of translation product was obtained when the target gene was fused with ibpA. However, in this case, most of the expressed protein was found in the insoluble fraction. In contrast, fusion partners such as S6 (30S ribosomal subunit S6) were found to enhance the expression level while keeping most of the translation products substantially soluble. Approximately 500 µg/ml of hBD-2 fusion protein was produced, 56% of which was soluble when S6 was fused with hBD-2. Furthermore, some of the examined fusion partners, including L7 (50S ribosomal subunit L7) and fkpB, were able to enhance the soluble expression of all three model proteins. Based on molar quantities, when compared to the native protein, the amount of soluble molecules was increased by approximately 19 – 32 fold.

### Parallel screening of optimal fusion partners for soluble expression of cytokine molecules

**Figure 4:**
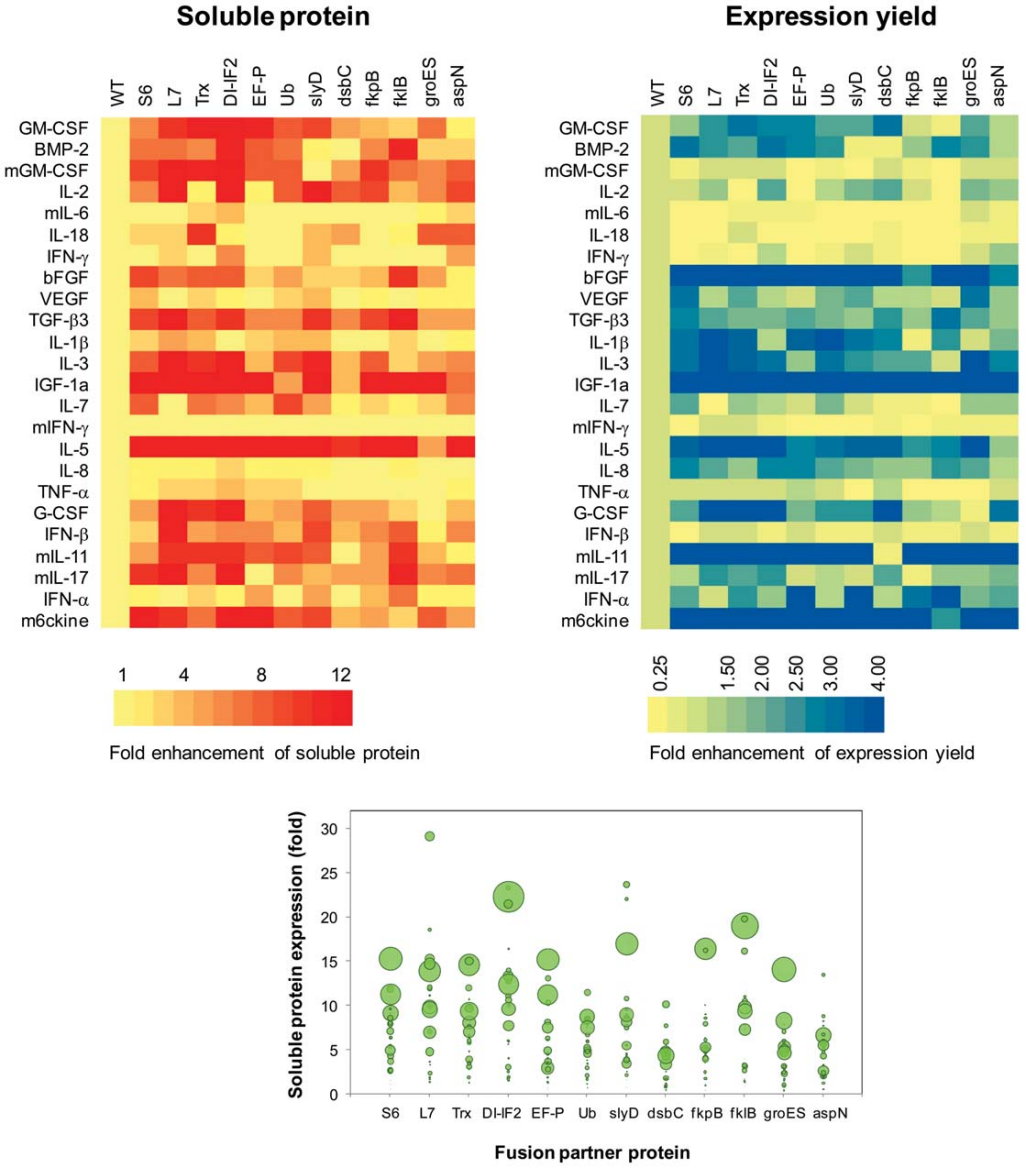
Combinatorial examination of fusion partners for the expression of different cytokines. (A) Fold enhancement of soluble expression of cytokines by the examined fusion partners. (B) Fold enhancement of total expression yield by fusion with the examined fusion partners. (C) A bubble chart where the size of each bubble diameter is proportional to the fold enhancement of the expression efficiency of total protein. Detailed stacked bar graphs of individual fusion protein are shown in Figure S1.

From the 88 tentative fusion partners examined above, we selected 12 fusion partners that gave rise to more than a five-fold increase in the amount of soluble products for at least two of the three target proteins (Figure 4 and Figure S1). It should be noted that only Trx was selected from the conventional fusion partners examined, while all other generally used fusion partners failed to improve the soluble expression of the primary model proteins substantially. While some of the conventional fusion partners greatly improved the solubility of the translation product, the total yield of the fusion protein was not enhanced as much (NTL9, Ub). In other cases, the partners did not increase the solubility, while the overall yield was improved (GST, NusA, Trx). In contrast, 11 fusion partners selected from the *E. coli* genome were able to improve the efficiency of gene expression while maintaining the translation product in a highly soluble form.

We next evaluated the effect of the selected fusion partners against 24 different cytokine species derived from humans and mice. When expressed from their native sequence, most of the examined cytokines exhibited very low yield and poor solubility (139 µg/ml and 18% average yield and solubility, respectively). However, upon fusion with the 12 selected fusion partners, most of the examined cytokines showed a substantially enhanced yield of soluble products due to increases in both total protein production and solubility (Figure 4). Among those, L7 and S6 were found to be exceptionally effective at enhancing the production of soluble proteins. Soluble yields of 20 out of 24 examined cytokines were improved when fused with the L7 protein with levels of enhancement ranging from 1.5 to 29 fold. Similarly, S6 increased the soluble expression of 21 cytokines from 2.5 to 15 fold.

### Relationship between solubility and physicochemical properties of protein

**Figure 5:**
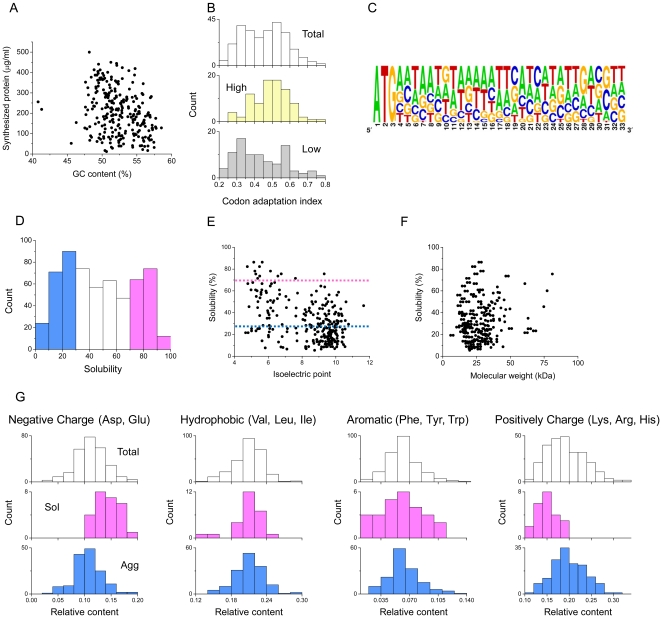
Statistical analysis for the relationships between solubility/yield and physicochemical properties. (A) Relationship between expression efficiency and GC content. (B) Histograms of codon adaptation index for highly expressed genes and poorly expressed genes. (C) Sequence logos of downstream region of proteins with high expression efficiency (>70% enhancement), which was created with WebLogo software [44]. (D) Solubility distribution for quantified proteins. Histogram of solubility for the quantified proteins in Figure 3. The proteins with solubilities <30% and >70% were defined as the aggregation-prone (Agg, colored blue) and soluble (Sol, colored pink) groups, respectively. Scatter plot of solubility versus isoelectric point (E) and molecular weight (F). Histograms of the relative contents of negatively charged residues (Asp and Glu) (Left), hydrophobic residues (Val, Leu and Ile), aromatic residues (Phe, Tyr, and Trp), and positively charged residues (Lys, Arg, and His) in the Total, Agg, and Sol groups.

The level of enhancement in the solubility and expression in response to fusion with the 12 fusion partners showed wide distributions depending on the targeted cytokine molecules. The set of expression and solubility data generated in this study (12×24 = 288) was analyzed for the presence of common properties of the nucleotide and amino acid sequences that determine the expression efficiency and solubility of the expressed fusion molecules. First, total expression levels of the examined constructs did not show clear correlations with their GC contents (Figure 5A). However, there appeared to be a positive correlation between protein expression efficiency and codon adaption index (CAI) (Figure 5B) as well as a certain degree of bias in the initial nucleotide sequence of the well-expressed fusion constructs (Figure 5C). Since all of the fusion partners were added at the N-terminus of the target proteins, this finding reflects the relative expression efficiency of the fusion partners due to the identities of their initial codons. The solubility data generated from the primary and secondary screening procedures were also analyzed to explore the general pattern correlating the physicochemical properties of the fusion proteins and their solubility. While the solubility of the fusion molecules appeared to be related to the composition of amino acids (for example, the contents of charged amino acids), the distribution of solubility generally seemed to occur at random against different parameters (Figure 5 D–G). Therefore, the effect of fusion partner appears to be due to the intrinsic nature of the fusion partners, rather than changes in the amino acid composition introduced by the fusion partners.

## Discussion

While the proteomics approach for understanding the networks of protein function is represented by characterization of global changes at the level of their expression/post-translation modification by mass spectrometry and 2D gel electrophoresis, data obtained from proteomic analysis frequently needs to be complemented with detailed information regarding the individual proteins participating in the functional networks, which can be accelerated using a reliable method for high-throughput expression and analysis of protein molecules. By programming with PCR-amplified genes, cell-free protein synthesis enables multiplexed, rapid preparation of protein molecules for subsequent downstream analyses such as structure determination and analysis of biological activities. With the use of automated liquid handling devices, thousands of recombinant proteins can be readily prepared for genome-wide expression analysis of ORFs, providing an ideal platform for ‘reverse proteomics’. In addition, unlike cell-based gene expression, the amounts of cell-free synthesized proteins can be precisely determined by measuring the incorporation of labeled amino acids into the synthesized proteins, allowing for precise quantification of total and soluble translation products.

However, the solubility issue of the expressed proteins remains the major hurdle to overcome for large-scale investigation of protein function. In this study, cell-free expression analysis of combinatorial fusion constructs between aggregation-prone target proteins and a series of fusion partners was used in an attempt to screen optimal fusion partners that provide the maximum expression level of soluble proteins. In addition to the commonly used generic fusion partners, we included highly abundant *E. coli* proteins in the list of tentative fusion partners. This was done for two reasons. First, although the primary purpose of adding fusion partners is to improve the solubility of target proteins, the presence of a fusion partner can also influence the efficiency of the expression of the entire fusion protein due to the initial codon effect. Therefore, by using the sequences of proteins that are highly expressed by the *E. coli* translational machinery, we expected to enhance the overall expression level of the target proteins in our cell-free synthesis system derived from *E. coli*. In addition, we assumed that highly abundant proteins have properties that enables them to decrease their aggregation, which will be necessary for bacterial cytoplasmic proteins to minimize their deposition at the concentrations required for their proper biological functions [33], [34]. Therefore, by using those abundant proteins as the fusion partners, we sought to enhance both the expression level and solubility of the resulting fusion proteins. Cytokines were selected as the target proteins since they are a growing group of proteins that act as mediators of cell-to-cell communication and thus have great potential for use as potential therapeutics as well as drug targets.

As expected, the *E. coli* proteins selected based on their abundance level were able to increase the soluble expression of the targeted cytokine proteins as well as the model proteins for primary screening. In this study, when several hundred fusion genes were systematically examined in parallel, different fusion partner proteins showed increased expression of soluble target proteins as well as the overall yield of expressed protein, with increases of as much as 29 fold and 15 fold, respectively, being observed in response to their fusion with aggregation-prone proteins. To understand the correlation between the sequence information and the expression efficiency/solubility in our fusion protein expression result, statistical analysis of fusion constructs was conducted. The results showed that AT nucleotides are biased in the initial region of highly expressed fusion genes. However, no significant correlation between physicochemical properties and the solubility of fused genes was observed. We also attempted to draw a common pattern of sequence-solubility relationship of the expressed fusion proteins using a computational sequence analysis algorithm (Table S5). However, again, we were not able to find meaningful correlations between the sequence properties obtained from the AGGRESCAN analysis and experimental results of protein solubility [35]. This might result from the difference between *in vivo* and *in vitro* environments for protein synthesis, which needs further investigation in the future.

Since the protein solubility varies significantly depending on the specific combination of fusion partner and target proteins, effective fusion partners for a given target protein should be determined empirically, which demands a high-throughput strategy for a large-scale gene fusion and protein expression system of fused gene constructs.

While the present study focused on the cell-free expression of aggregation prone proteins, we expect that the results presented herein can be extended to cell-based protein expression for large scale production of specific target proteins since the present cell-free protein synthesis system mimics the cytoplasmic conditions of the *E. coli* cells. Since most of the fusion partners screened in this study are ribosomal subunit proteins, the possibility that their use in cell-based gene expression can interfere with the assembly of endogenous ribosomes cannot be excluded; nevertheless, they could be engineered to be incapable of participating in ribosome assembly. Furthermore, the approach presented herein will be applicable to various fields involving global expression and analysis of various genomic resources.

## Supporting Information

Figure S1 Expression yield and solubility of cytokines fused with 12 fusion partners. 24 cytokine genes that otherwise exhibit poor expression level and solubility were fused by PCR with 12 fusion partners selected from the initial screening. The fusion constructs were incubated in a cell-free protein synthesis system and analyzed for their final expression level and solubility as described in Materials and Methods.
(DOC)

Table S1 Primers used in this study.
(DOC)

Table S2 Solubility and total expression yield of BD2.
(DOC)

Table S3 Solubility and total expression yield of EGF.
(DOC)

Table S4 Solubility and total expression yield of EPO.
(DOC)

Table S5 AGGRESCAN analysis.
(DOC)
