# Supplementary material for: Expression Screening of Fusion Partners from an E. coli Genome for Soluble Expression of Recombinant Proteins in a Cell-Free Protein Synthesis System
Source: PLoS One. 2011 Nov 2;6(11):e26875. doi: 10.1371/journal.pone.0026875 (PMC3206877; doi:10.1371/journal.pone.0026875)
Supplement: Figure S1 — Expression yield and solubility of cytokines fused with 12 fusion partners. 24 cytokine genes that otherwise exhibit poor expression level and solubility were fused by PCR with 12 fusion partners selected from the initial screening. The fusion constructs were incubated in a cell-free protein synthesis system and analyzed for their final expression level and solubility as described in Materials and Methods. (DOC) [file pone.0026875.s001.doc]

**Figure S1**. Expression yield and solubility of cytokines fused with 12 fusion partners.
