# Supplementary material for: Expression Screening of Fusion Partners from an E. coli Genome for Soluble Expression of Recombinant Proteins in a Cell-Free Protein Synthesis System
Source: PLoS One. 2011 Nov 2;6(11):e26875. doi: 10.1371/journal.pone.0026875 (PMC3206877; doi:10.1371/journal.pone.0026875)
Supplement: Table S1 — Primers used in this study. (DOC) [file pone.0026875.s002.doc]

Table S1. Primers used in this study

| **Primer** | **Sequence (5’🡪3’)** |
| --- | --- |
| P1-FP-MBP | aagaaggagatatacatatgaaaatcgaagaaggtaaact |
| P2-FP-MBP | GCGGCCTTCGATactaccacccttggtgatacgagtctgcg |
| P1-FP-NusA | aagaaggagatatacatatgaacaaagaaattttggctgt |
| P2-FP-NusA | GCGGCCTTCGATactaccacccgcttcgtcaccgaaccagc |
| P1-FP-GST | aagaaggagatatacatatgtcccctatactaggttattg |
| P2-FP-GST | GCGGCCTTCGATactaccaccttttggaggatggtcgccac |
| P1-FP-Trx | aagaaggagatatacatatgagcgataaaattattcacct |
| P2-FP-Trx | GCGGCCTTCGATactaccacccgccaggttagcgtcgagga |
| P1-FP-Ubiquitin | aagaaggagatatacatatgCAGATTTTCGTCAAGACCCT |
| P2-FP-Ubiquitin | GCGGCCTTCGATactaccaccACCACCTCTTAGCCTTAGGA |
| P1-FP-DI-IF2 | aagaaggagatatacatatgacagatgtaacgattaaaac |
| P2-FP-DI-IF2 | GCGGCCTTCGATactaccacccactttgtctttttccgcag |
| P1-FP-NTL9 | aagaaggagatatacatatgcaagttattctgcttgataa |
| P2-FP-NTL9 | GCGGCCTTCGATactaccaccagcttccagttcagcgcgac |
| P1-RS-S1 | aagaaggagatatacatatgactgaatcttttgctcaact |
| P2-RS-S1 | GCGGCCTTCGATactaccaccctcgcctttagctgctttga |
| P1-RS-S2 | aagaaggagatatacatatggcaactgtttccatgcgcga |
| P2-RS-S2 | GCGGCCTTCGATactaccaccctcagcttctacgaagcttt |
| P1-RS-S3 | aagaaggagatatacatatgggtcagaaagtacatcctaa |
| P2-RS-S3 | GCGGCCTTCGATactaccacctttacggcctttacgctgct |
| P1-RS-S4 | aagaaggagatatacatatggcaagatatttgggtcctaa |
| P2-RS-S4 | GCGGCCTTCGATactaccacccttggagtaaagctcgacga |
| P1-RS-S5 | aagaaggagatatacatatggctcacatcgaaaaacaagc |
| P2-RS-S5 | GCGGCCTTCGATactaccacctttccccagaatttcttcaa |
| P1-RS-S6 | aagaaggagatatacatatgcgtcattacgaaatcgtttt |
| P2-RS-S6 | GCGGCCTTCGATactaccaccctcttcagaatccccagctt |
| P1-RS-S7 | aagaaggagatatacatatgccacgtcgtcgcgtcattgg |
| P2-RS-S7 | GCGGCCTTCGATactaccaccatttaagtagcccaaagcgg |
| P1-RS-S8 | aagaaggagatatacatatgagcatgcaagatccgatcgc |
| P2-RS-S8 | GCGGCCTTCGATactaccaccggctacgtagcagataatttc |
| P1-RS-S9 | aagaaggagatatacatatggctgaaaatcaatactacgg |
| P2-RS-S9 | GCGGCCTTCGATactaccaccacgtttggagaactgcggac |
| P1-RS-S10 | aagaaggagatatacatatgcagaaccaaagaatccgtat |
| P2-RS-S10 | GCGGCCTTCGATactaccaccacccaggctgatctgcacgt |
| P1-RS-S11 | aagaaggagatatacatatggcaaaggcaccaattcgtgc |
| P2-RS-S11 | GCGGCCTTCGATactaccacctacgcgacgttttttcggcg |
| P1-RS-S12 | aagaaggagatatacatatggcaacagttaaccagctggt |
| P2-RS-S12 | GCGGCCTTCGATactaccaccagccttaggacgcttcacgc |
| P1-RS-S13 | aagaaggagatatacatatggcccgtatagcaggcattaa |
| P2-RS-S13 | GCGGCCTTCGATactaccacctttcttgatcggtttgcgcg |
| P1-RS-S14 | aagaaggagatatacatatggctaagcaatcaatgaaagc |
| P2-RS-S14 | GCGGCCTTCGATactaccaccccagctagcctttttcagac |
| P1-RS-S15 | aagaaggagatatacatatgtctctaagtactgaagcaac |
| P2-RS-S15 | GCGGCCTTCGATactaccaccgcgacgcagacccaggcgct |
| P1-RS-S16 | aagaaggagatatacatatggtaactattcgtttagcacg |
| P2-RS-S16 | GCGGCCTTCGATactaccaccagctgctttgtttacttctt |
| P1-RS-S17 | aagaaggagatatacatatgaccgataaaatccgtactct |
| P2-RS-S17 | GCGGCCTTCGATactaccacccagaaccgctttctctacaa |
| P1-RS-S18 | aagaaggagatatacatatggcacgttatttccgtcgtcg |
| P2-RS-S18 | GCGGCCTTCGATactaccaccctgatggcgatcagtgtacg |
| P1-RS-S19 | aagaaggagatatacatatgccacgttctctcaagaaagg |
| P2-RS-S19 | GCGGCCTTCGATactaccacctttcttcttcgcttttttat |
| P1-RS-S20 | aagaaggagatatacatatggctaatatcaaatcagctaa |
| P2-RS-S20 | GCGGCCTTCGATactaccaccagccagtttgttgatctgtg |
| P1-RS-S21 | aagaaggagatatacatatgccggtaattaaagtacgtga |
| P2-RS-S21 | GCGGCCTTCGATactaccaccgtacagacgagtgcggcgtg |
| P1-RS-S22 | aagaaggagatatacatatgaaatcgaaccgtcaggcacg |
| P2-RS-S22 | GCGGCCTTCGATactaccacccttttcagcggggcgttttct |
| P1-RL-L1 | aagaaggagatatacatatggctaaactgaccaagcgcat |
| P2-RL-L1 | GCGGCCTTCGATactaccaccgtttacagaagcgctcaggc |
| P1-RL-L2 | aagaaggagatatacatatggcagttgttaaatgtaaacc |
| P2-RL-L2 | GCGGCCTTCGATactaccacctttgctacggcgacgtacga |
| P1-RL-L3 | aagaaggagatatacatatgattggtttagtcggtaaaaa |
| P2-RL-L3 | GCGGCCTTCGATactaccacccgccttcacagctggtttaa |
| P1-RL-L4 | aaggagatatacatatggaattagtattgaaagacgc |
| P2-RL-L4 | GCGGCCTTCGATactaccacctgccagcatctcctcaactt |
| P1-RL-L5 | aagaaggagatatacatatggcgaaactgcatgattacta |
| P2-RL-L5 | GCGGCCTTCGATactaccacccttgcggaacgggaagtcaa |
| P1-RL-L6 | aagaaggagatatacatatgtctcgtgttgctaaagcacc |
| P2-RL-L6 | GCGGCCTTCGATactaccacccttcttcttagcctctttgg |
| P1-RL-L7 | aagaaggagatatacatatgtctatcactaaagatcaaat |
| P2-RL-L7 | GCGGCCTTCGATactaccacctttaacttcaacttcagcgc |
| P1-RL-L9 | aagaaggagatatacatatgcaagttattctgcttgataa |
| P2-RL-L9 | GCGGCCTTCGATactaccaccttcagctactacgtttacga |
| P1-RL-L10 | aagaaggagatatacatatggctttaaatcttcaagacaa |
| P2-RL-L10 | GCGGCCTTCGATactaccaccagcagcttctttcgcatcgc |
| P1-RL-L11 | aagaaggagatatacatatggctaagaaagtacaagccta |
| P2-RL-L11 | GCGGCCTTCGATactaccaccgtcctccactaccaggccca |
| P1-RL-L13 | aagaaggagatatacatatgaaaacttttacagctaaacc |
| P2-RL-L13 | GCGGCCTTCGATactaccaccgatgtcaagaacttgcggtt |
| P1-RL-L14 | aagaaggagatatacatatgatccaagaacagactatgct |
| P2-RL-L14 | GCGGCCTTCGATactaccaccgagtacttctggtgccagag |
| P1-RL-L15 | aagaaggagatatacatatgcgtttaaatactctgtctcc |
| P2-RL-L15 | GCGGCCTTCGATactaccaccttcctcgattttaccgccag |
| P1-RL-L16 | aagaaggagatatacatatgttacaaccaaagcgtacaaa |
| P2-RL-L16 | GCGGCCTTCGATactaccacccatcaccgtcttagttacaa |
| P1-RL-L17 | aagaaggagatatacatatgcgccatcgtaagagtggtcg |
| P2-RL-L17 | GCGGCCTTCGATactaccaccctctgcagcagcttctgctt |
| P1-RL-L18 | aagaaggagatatacatatggataagaaatctgctcgtat |
| P2-RL-L18 | GCGGCCTTCGATactaccaccgaactgaaggccagcttcac |
| P1-RL-L19 | aagaaggagatatacatatgagcaacattattaagcaact |
| P2-RL-L19 | GCGGCCTTCGATactaccaccgttaagacgctctttgatac |
| P1-RL-L20 | aagaaggagatatacatatggctcgcgtaaaacgtggtgt |
| P2-RL-L20 | GCGGCCTTCGATactaccacctgccagagctgctttcgctt |
| P1-RL-L21 | aagaaggagatatacatatgtacgcggttttccaaagtgg |
| P2-RL-L21 | GCGGCCTTCGATactaccaccggcgctgatgccagtaattt |
| P1-RL-L22 | aagaaggagatatacatatggaaactatcgctaaacatcg |
| P2-RL-L22 | GCGGCCTTCGATactaccaccgcgatcggacacaaccacag |
| P1-RL-L23 | aagaaggagatatacatatgattcgtgaagaacgtctgct |
| P2-RL-L23 | GCGGCCTTCGATactaccaccctcagcgccgccaacgaagt |
| P1-RL-L24 | aagaaggagatatacatatggcagcgaaaatccgtcgtga |
| P2-RL-L24 | GCGGCCTTCGATactaccacccttgatagtttcgctgttag |
| P1-RL-L25 | aagaaggagatatacatatgtttactatcaacgcagaagt |
| P2-RL-L25 | GCGGCCTTCGATactaccaccagcgcgaacgaagtcgatgt |
| P1-RL-L27 | aagaaggagatatacatatggcacataaaaaggctggcgg |
| P2-RL-L27 | GCGGCCTTCGATactaccaccttcagcttcgatgctgataa |
| P1-RL-L28 | aagaaggagatatacatatgtcccgagtctgccaagttac |
| P2-RL-L28 | GCGGCCTTCGATactaccaccgtacttttcgccacgggcac |
| P1-RL-L29 | aagaaggagatatacatatgaaagcaaaagagctgcgtga |
| P2-RL-L29 | GCGGCCTTCGATactaccacccgcacccgccttctcgttca |
| P1-RL-L30 | aagaaggagatatacatatggcaaagactattaaaattac |
| P2-RL-L30 | GCGGCCTTCGATactaccaccctcctcaactttaaccatga |
| P1-RL-L31B | aagaaggagatatacatatgaagcccaatatccatcctga |
| P2-RL-L31B | GCGGCCTTCGATactaccacccgccccctttttcgtgctaa |
| P1-RL-L31 | aagaaggagatatacatatgaaaaaagatattcacccgaa |
| P2-RL-L31 | GCGGCCTTCGATactaccacctttgctgcccgggatgttga |
| P1-RL-L32 | aagaaggagatatacatatggccgtacaacagaataaacc |
| P2-RL-L32 | GCGGCCTTCGATactaccacccttagcgatgaccttgcggc |
| P1-RL-L33 | aagaaggagatatacatatggctaaaggtattcgtgagaa |
| P2-RL-L33 | GCGGCCTTCGATactaccacctttgattttcgcttctttgt |
| P1-RL-L34 | aagaaggagatatacatatgatgaaacgcacttttcaacc |
| P2-RL-L34 | GCGGCCTTCGATactaccacccttagaaacggtcagacgag |
| P1-RL-L35 | aagaaggagatatacatatgccaaaaattaagaccgtacg |
| P2-RL-L35 | GCGGCCTTCGATactaccacctgcgtacggcaggcacgcga |
| P1-RL-L36 | aagaaggagatatacatatgaaagttcgtgcttccgtcaa |
| P2-RL-L36 | GCGGCCTTCGATactaccaccgccttggcgctgtttatgct |
| P1-FP-ibpA | aagaaggagatatacatatgcgtaactttgatttatcccc |
| P2-FP-ibpA | GCGGCCTTCGATactaccaccgttgatttcgatacggcgcg |
| P1-FP-ibpB | aagaaggagatatacatatgcgtaacttcgatttatcccc |
| P2-FP-ibpB | GCGGCCTTCGATactaccaccgctatttaacgcgggacgtt |
| P1-FP-skp | aagaaggagatatacatatgaaaaagtggttattagctgc |
| P2-FP-skp | GCGGCCTTCGATactaccacctttaacctgtttcagtacgt |
| P1-FP-slyD | aagaaggagatatacatatgaaagtagcaaaagacctggt |
| P2-FP-slyD | GCGGCCTTCGATactaccaccgtggcaaccgcaaccgccgt |
| P1-FP-dsbA | aagaaggagatatacatatgaaaaagatttggctggcgct |
| P2-FP-dsbA | GCGGCCTTCGATactaccaccttttttctcggacagatatt |
| P1-FP-dsbB | aagaaggagatatacatatgttgcgatttttgaaccaatg |
| P2-FP-dsbB | GCGGCCTTCGATactaccaccgcgaccgaacagatcacgtt |
| P1-FP-dsbC | aagaaggagatatacatatgaagaaaggttttatgttgtt |
| P2-FP-dsbC | GCGGCCTTCGATactaccacctttaccgctggtcatttttt |
| P1-FP-secB | aagaaggagatatacatatgtcagaacaaaacaacactga |
| P2-FP-secB | GCGGCCTTCGATactaccaccggcatcctgatgttcttcag |
| P1-FP-secE | aagaaggagatatacatatgagtgcgaataccgaagctca |
| P2-FP-secE | GCGGCCTTCGATactaccaccgaacctcaggccagtgataa |
| P1-FP-secG | aagaaggagatatacatatgtatgaagctcttttagtagt |
| P2-FP-secG | GCGGCCTTCGATactaccaccgttcgggatatcgctggtcg |
| P1-FP-grpE | aagaaggagatatacatatgagtagtaaagaacagaaaac |
| P2-FP-grpE | GCGGCCTTCGATactaccaccagcttttgctttcgctacag |
| P1-FP-fkpB | aagaaggagatatacatatgtctgaatctgtacagagcaa |
| P2-FP-fkpB | GCGGCCTTCGATactaccacccgcctccagtgccggatcga |
| P1-FP-fklB | aagaaggagatatacatatgaccaccccaacttttgacac |
| P2-FP-fklB | GCGGCCTTCGATactaccaccgaggatttccagcagttcga |
| P1-FP-groEL | aagaaggagatatacatatggcagctaaagacgtaaaatt |
| P2-FP-groEL | GCGGCCTTCGATactaccacccatcatgccgcccatgccac |
| P1-FP-groES | aagaaggagatatacatatgaatattcgtccattgcatga |
| P2-FP-groES | GCGGCCTTCGATactaccacccgcttcaacaattgccagaa |
| P1-groEL191-345 | aagaaggagatatacatatggaaggtatgcagttcgaccg |
| P2-groEL191-345 | GCGGCCTTCGATactaccaccacggccctggattgcagctt |
| P2-groEL191-376 | GCGGCCTTCGATactaccaccaacgccgcctgccagtttcg |
| P1-FP-lysN | aagaaggagatatacatatgtctgaacaacacgcacaggg |
| P2-FP-lysN | GCGGCCTTCGATactaccacccagacgcaactcggtgcagt |
| P1-FP-aspN | aagaaggagatatacatatgcgtacagaatattgtggaca |
| P2-FP-aspN | GCGGCCTTCGATactaccaccgatgatagtcagcgaggacg |
| P1-FP-asnN | aagaaggagatatacatatgagcgttgtgcctgtagccga |
| P2-FP-asnN | GCGGCCTTCGATactaccaccaacttcaaccttgctggcct |
| P3-fusion-EGF | ggtggtagtATCGAAGGCCGCaatagtgactctgaatgtcc |
| P4-fusion-EGF | TTAATGATGATGATGATGATGgcgcagttcccaccacttca |
| P3-fusion-BD2 | ggtggtagtATCGAAGGCCGCggtattggcgatccggttac |
| P4-fusion-BD2 | TTAATGATGATGATGATGATGtggctttttgcagcatttcg |
| P3-fusion-EPO | ggtggtagtATCGAAGGCCGCGCCCCACCACGCCTCATCTG |
| P4-fusion-EPO | TTAATGATGATGATGATGATGTCTGTCCCCTGTCCTGCAGG |
| P3-GMCSF-G1 | ggtggtagtATCGAAGGCCGCgcacccgcccgctcgcccag |
| P4-GMCSF-G1 | ttaatgatgatgatgatgatgctcctggactggctcccagc |
| P3-BMP-2-G4 | ggtggtagtATCGAAGGCCGCcaagccaaacacaaacagcg |
| P4-BMP-2-G4 | ttaatgatgatgatgatgatggcgacacccacaaccctcca |
| P3-mGM-CSF-G6 | ggtggtagtATCGAAGGCCGCgcacccacccgctcacccat |
| P4-mGM-CSF-G6 | TTAATGATGATGATGATGATGtttttggactggttttttgc |
| P3-IL-2-G9 | ggtggtagtATCGAAGGCCGCgcacctacttcaagttctac |
| P4-IL-2-G9 | ttaatgatgatgatgatgatgagttagtgttgagatgatgc |
| P3-mIL-6-G15 | ggtggtagtATCGAAGGCCGCttccctacttcacaagtccg |
| P4-mIL-6-G15 | TTAATGATGATGATGATGATGggtttgccgagtagatctca |
| P3-IL-18-G23 | ggtggtagtATCGAAGGCCGCtactttggcaagcttgaatc |
| P4-IL-18-G23 | TTAATGATGATGATGATGATGgtcttcgttttgaacagtga |
| P3-IFN-G-G27 | ggtggtagtATCGAAGGCCGCcaggacccatatgtacaaga |
| P4-IFN-G-G27 | TTAATGATGATGATGATGATGctgggatgctcttcgacctc |
| P3-VEGF-G39 | ggtggtagtATCGAAGGCCGCgcacccatggcagaaggagg |
| P4-VEGF-G39 | ttaatgatgatgatgatgatgccgcctcggcttgtcacatc |
| P3-TGF-B3-G65 | ggtggtagtATCGAAGGCCGCgctttggacaccaattactg |
| P4-TGF-B3-G65 | ttaatgatgatgatgatgatggctacatttacaagacttca |
| P3-IL-1b-G76 | ggtggtagtATCGAAGGCCGCgcacctgtacgatcactgaa |
| P4-IL-1b-G76 | ttaatgatgatgatgatgatgggaagacacaaattgcatgg |
| P3-IL-3-G81 | ggtggtagtATCGAAGGCCGCgctcccatgacccagacaac |
| P4-IL-3-G81 | TTAATGATGATGATGATGATGaaagatcgcgaggctcaaag |
| P3-IGF-1a-G83 | ggtggtagtATCGAAGGCCGCggaccggagacgctctgcgg |
| P4-IGF-1a-G83 | TTAATGATGATGATGATGATGcatcctgtagttcttgtttc |
| P3-IL-7-G93 | ggtggtagtATCGAAGGCCGCgattgtgatattgaaggtaa |
| P4-IL-7-G93 | TTAATGATGATGATGATGATGgtgttctttagtgcccatca |
| P3-mIFN-G-G96 | ggtggtagtATCGAAGGCCGCcacggcacagtcattgaaag |
| P4-mIFN-G-G96 | TTAATGATGATGATGATGATGgcagcgactccttttccgct |
| P3-IL-5-G102 | ggtggtagtATCGAAGGCCGCatccccacagaaattcccac |
| P4-IL-5-G102 | ttaatgatgatgatgatgatgactttctattatccactcgg |
| P3-IL-8-G112 | ggtggtagtATCGAAGGCCGCagtgctaaagaacttagatg |
| P4-IL-8-G112 | ttaatgatgatgatgatgatgtgaattctcagccctcttca |
| P3-TNF-A-G116 | ggtggtagtATCGAAGGCCGCgtcagatcatcttctcgaac |
| P4-TNF-A-G116 | ttaatgatgatgatgatgatgcagggcaatgatcccaaagt |
| P3-G-CSF-G118 | ggtggtagtATCGAAGGCCGCacccccctgggccctgccag |
| P4-G-CSF-G118 | ttaatgatgatgatgatgatggggctgggcaaggtggcgta |
| P3-IFN-B-G120 | ggtggtagtATCGAAGGCCGCagctacaacttgcttggatt |
| P4-IFN-B-G120 | TTAATGATGATGATGATGATGgtttcggaggtaacctgtaa |
| P3-mIL-11-G133 | ggtggtagtATCGAAGGCCGCcctgggccaccagctggctc |
| P4-mIL-11-G133 | TTAATGATGATGATGATGATGcagtcgagtctttaacaaca |
| P3-mIL-17-G135 | ggtggtagtATCGAAGGCCGCgcagcgatcatccctcaaag |
| P4-mIL-17-G135 | TTAATGATGATGATGATGATGggctgcctggcggacaatcg |
| P3-IFN-A-G136 | ggtggtagtATCGAAGGCCGCtgtgatctccctgagaccca |
| P4-IFN-A-G136 | ttaatgatgatgatgatgatgttccttcctccttaatcttt |
| P3-m6ckine-G147 | ggtggtagtATCGAAGGCCGCagtgatggagggggtcagga |
| P4-m6ckine-G147 | TTAATGATGATGATGATGATGtcctcttgagggctgtgtct |
