# Supplementary material for: Expression Screening of Fusion Partners from an E. coli Genome for Soluble Expression of Recombinant Proteins in a Cell-Free Protein Synthesis System
Source: PLoS One. 2011 Nov 2;6(11):e26875. doi: 10.1371/journal.pone.0026875 (PMC3206877; doi:10.1371/journal.pone.0026875)
Supplement: Table S2 — Solubility and total expression yield of BD2. (DOC) [file pone.0026875.s003.doc]

**Table S2**. Solubility and total expression yield of BD2.

| **BD2** | **Soluble**  **(μg/ml)** | **Insoluble**  **(μg/ml)** | **Total**  **(μg/ml)** | **Solubility**  **(%)** |
| --- | --- | --- | --- | --- |
| WT | 12 | 55 | 67 | 18 |
| S1 | 21 | 33 | 54 | 39 |
| S2 | 98 | 109 | 207 | 47 |
| S3 | 63 | 234 | 298 | 21 |
| S4 | 84 | 113 | 197 | 43 |
| S5 | 62 | 81 | 143 | 44 |
| S6 | 368 | 76 | 444 | 83 |
| S7 | 19 | 66 | 85 | 23 |
| S8 | 141 | 157 | 298 | 47 |
| S9 | 28 | 165 | 193 | 15 |
| S10 | 142 | 177 | 319 | 44 |
| S11 | 57 | 131 | 188 | 30 |
| S12 | 41 | 148 | 188 | 22 |
| S13 | 50 | 78 | 128 | 39 |
| S14 | 46 | 37 | 83 | 55 |
| S15 | 77 | 29 | 106 | 73 |
| S16 | 134 | 90 | 224 | 60 |
| S17 | 186 | 84 | 270 | 69 |
| S18 | 29 | 89 | 118 | 25 |
| S19 | 47 | 32 | 79 | 60 |
| S20 | 89 | 45 | 134 | 66 |
| S21 | 58 | 28 | 86 | 67 |
| S22 | 99 | 24 | 123 | 81 |
| L1 | 48 | 171 | 218 | 22 |
| L2 | 57 | 87 | 144 | 40 |
| L3 | 118 | 248 | 366 | 32 |
| L4 | 25 | 66 | 90 | 27 |
| L5 | 34 | 331 | 365 | 9 |
| L6 | 169 | 103 | 272 | 62 |
| L7 | 310 | 42 | 352 | 88 |
| L9 | 178 | 218 | 395 | 45 |
| L10 | 35 | 243 | 279 | 13 |
| L11 | 220 | 88 | 308 | 71 |
| L13 | 120 | 236 | 357 | 34 |
| L14 | 29 | 271 | 300 | 10 |
| L15 | 25 | 154 | 179 | 14 |
| L16 | 69 | 120 | 189 | 36 |
| L17 | 141 | 60 | 201 | 70 |
| L18 | 45 | 168 | 213 | 21 |
| L19 | 130 | 199 | 329 | 39 |
| L20 | 37 | 111 | 148 | 25 |
| L21 | 27 | 360 | 387 | 7 |
| L22 | 66 | 155 | 222 | 30 |
| L23 | 42 | 280 | 322 | 13 |
| L24 | 133 | 60 | 193 | 69 |
| L25 | 146 | 97 | 243 | 60 |
| L27 | 37 | 70 | 106 | 35 |
| L28 | 28 | 110 | 138 | 20 |
| L29 | 51 | 23 | 73 | 69 |
| L30 | 77 | 46 | 123 | 62 |
| L31 | 29 | 26 | 55 | 52 |
| L31B | 71 | 57 | 128 | 55 |
| L32 | 46 | 23 | 69 | 67 |
| L33 | 32 | 20 | 52 | 62 |
| L34 | 22 | 12 | 34 | 64 |
| L35 | 10 | 7 | 17 | 56 |
| L36 | 34 | 27 | 61 | 56 |
| MBP | 127 | 31 | 159 | 80 |
| Trx | 252 | 34 | 286 | 88 |
| GST | 218 | 38 | 256 | 85 |
| NusA | 100 | 26 | 126 | 80 |
| Ub | 183 | 32 | 215 | 85 |
| DI-IF2 | 407 | 43 | 450 | 90 |
| EF-Tu | 120 | 304 | 424 | 28 |
| EF-P | 297 | 115 | 413 | 72 |
| IF1 | 198 | 52 | 250 | 79 |
| IF3 | 246 | 61 | 308 | 80 |
| NTL9 | 57 | 24 | 82 | 70 |
| ibpA | 25 | 475 | 500 | 5 |
| ibpB | 73 | 368 | 440 | 17 |
| skp | 126 | 242 | 368 | 34 |
| slyD | 315 | 36 | 351 | 90 |
| dsbA | 156 | 65 | 221 | 71 |
| dsbB | 22 | 334 | 356 | 6 |
| dsbC | 189 | 224 | 413 | 46 |
| secB | 219 | 73 | 292 | 75 |
| secE | 25 | 245 | 270 | 9 |
| secG | 21 | 116 | 137 | 15 |
| grpE | 247 | 32 | 278 | 89 |
| fkpB | 243 | 33 | 276 | 88 |
| fklB | 361 | 58 | 419 | 86 |
| groEL | 26 | 13 | 39 | 68 |
| groES | 218 | 178 | 396 | 55 |
| groEL191-345 | 49 | 16 | 65 | 75 |
| groEL191-376 | 30 | 9 | 39 | 77 |
| lysN | 182 | 73 | 255 | 71 |
| aspN | 188 | 101 | 289 | 65 |
| asnN | 80 | 31 | 112 | 72 |
