# Supplementary material for: Expression Screening of Fusion Partners from an E. coli Genome for Soluble Expression of Recombinant Proteins in a Cell-Free Protein Synthesis System
Source: PLoS One. 2011 Nov 2;6(11):e26875. doi: 10.1371/journal.pone.0026875 (PMC3206877; doi:10.1371/journal.pone.0026875)
Supplement: Table S3 — Solubility and total expression yield of EGF. (DOC) [file pone.0026875.s004.doc]

**Table S3**. Solubility and total expression yield of EGF.

| **EGF** | **Soluble**  **(μg/ml)** | **Insoluble**  **(μg/ml)** | **Total**  **(μg/ml)** | **Solubility**  **(%)** |
| --- | --- | --- | --- | --- |
| WT | 16 | 67 | 83 | 19 |
| S1 | 17 | 56 | 73 | 23 |
| S2 | 56 | 171 | 227 | 25 |
| S3 | 64 | 275 | 339 | 19 |
| S4 | 37 | 239 | 276 | 13 |
| S5 | 35 | 192 | 227 | 15 |
| S6 | 247 | 188 | 435 | 57 |
| S7 | 19 | 152 | 171 | 11 |
| S8 | 77 | 221 | 298 | 26 |
| S9 | 37 | 248 | 285 | 13 |
| S10 | 119 | 290 | 409 | 29 |
| S11 | 46 | 215 | 261 | 18 |
| S12 | 33 | 216 | 249 | 13 |
| S13 | 45 | 122 | 167 | 27 |
| S14 | 39 | 182 | 221 | 18 |
| S15 | 41 | 114 | 155 | 26 |
| S16 | 71 | 239 | 310 | 23 |
| S17 | 117 | 184 | 301 | 39 |
| S18 | 30 | 187 | 217 | 14 |
| S19 | 22 | 50 | 72 | 31 |
| S20 | 55 | 198 | 253 | 22 |
| S21 | 72 | 121 | 193 | 37 |
| S22 | 50 | 39 | 89 | 56 |
| L1 | 34 | 136 | 170 | 20 |
| L2 | 55 | 113 | 168 | 33 |
| L3 | 75 | 177 | 252 | 30 |
| L4 | 14 | 88 | 102 | 14 |
| L5 | 28 | 287 | 315 | 9 |
| L6 | 78 | 242 | 320 | 24 |
| L7 | 220 | 105 | 325 | 68 |
| L9 | 86 | 299 | 385 | 22 |
| L10 | 30 | 322 | 352 | 9 |
| L11 | 150 | 209 | 359 | 42 |
| L13 | 90 | 311 | 401 | 22 |
| L14 | 28 | 297 | 325 | 9 |
| L15 | 25 | 208 | 233 | 11 |
| L16 | 22 | 227 | 249 | 9 |
| L17 | 35 | 55 | 90 | 39 |
| L18 | 34 | 224 | 258 | 13 |
| L19 | 116 | 246 | 362 | 32 |
| L20 | 52 | 125 | 177 | 29 |
| L21 | 31 | 318 | 349 | 9 |
| L22 | 42 | 273 | 315 | 13 |
| L23 | 48 | 357 | 405 | 12 |
| L24 | 79 | 211 | 290 | 27 |
| L25 | 49 | 182 | 231 | 21 |
| L27 | 56 | 131 | 187 | 30 |
| L28 | 53 | 163 | 216 | 25 |
| L29 | 55 | 190 | 245 | 22 |
| L30 | 72 | 106 | 178 | 40 |
| L31 | 26 | 51 | 77 | 34 |
| L31B | 39 | 177 | 216 | 18 |
| L32 | 73 | 113 | 186 | 39 |
| L33 | 43 | 111 | 154 | 28 |
| L34 | 13 | 15 | 28 | 46 |
| L35 | 6 | 6 | 12 | 50 |
| L36 | 26 | 71 | 97 | 27 |
| MBP | 53 | 19 | 72 | 74 |
| Trx | 186 | 74 | 260 | 72 |
| GST | 62 | 157 | 219 | 28 |
| NusA | 41 | 149 | 190 | 22 |
| Ub | 143 | 79 | 222 | 64 |
| DI-IF2 | 286 | 92 | 378 | 76 |
| EF-Tu | 94 | 276 | 370 | 25 |
| EF-P | 167 | 207 | 374 | 45 |
| IF1 | 82 | 73 | 155 | 53 |
| IF3 | 167 | 166 | 333 | 50 |
| NTL9 | 82 | 152 | 234 | 35 |
| ibpA | 29 | 348 | 377 | 8 |
| ibpB | 53 | 320 | 372 | 14 |
| skp | 108 | 264 | 372 | 29 |
| slyD | 240 | 38 | 278 | 86 |
| dsbA | 94 | 141 | 235 | 40 |
| dsbB | 23 | 321 | 343 | 7 |
| dsbC | 172 | 216 | 387 | 44 |
| secB | 185 | 92 | 277 | 67 |
| secE | 20 | 260 | 280 | 7 |
| secG | 33 | 151 | 184 | 18 |
| grpE | 175 | 112 | 288 | 61 |
| fkpB | 216 | 46 | 262 | 82 |
| fklB | 303 | 85 | 387 | 78 |
| groEL | 22 | 67 | 89 | 25 |
| groES | 104 | 262 | 366 | 28 |
| groEL191-345 | 31 | 25 | 56 | 55 |
| groEL191-376 | 25 | 16 | 41 | 61 |
| lysN | 101 | 165 | 266 | 38 |
| aspN | 145 | 145 | 290 | 50 |
| asnN | 61 | 41 | 102 | 60 |
