# Supplementary material for: Expression Screening of Fusion Partners from an E. coli Genome for Soluble Expression of Recombinant Proteins in a Cell-Free Protein Synthesis System
Source: PLoS One. 2011 Nov 2;6(11):e26875. doi: 10.1371/journal.pone.0026875 (PMC3206877; doi:10.1371/journal.pone.0026875)
Supplement: Table S4 — Solubility and total expression yield of EPO. (DOC) [file pone.0026875.s005.doc]

**Table S4.** Solubility and total expression yield of EPO.

| **EPO** | **Soluble**  **(μg/ml)** | **Insoluble**  **(μg/ml)** | **Total**  **(μg/ml)** | **Solubility**  **(%)** |
| --- | --- | --- | --- | --- |
| WT | 7 | 121 | 128 | 5 |
| S1 | 37 | 12 | 49 | 76 |
| S2 | 39 | 52 | 91 | 43 |
| S3 | 49 | 106 | 155 | 32 |
| S4 | 46 | 148 | 194 | 24 |
| S5 | 26 | 72 | 98 | 27 |
| S6 | 77 | 108 | 185 | 42 |
| S7 | 29 | 158 | 187 | 16 |
| S8 | 40 | 45 | 85 | 47 |
| S9 | 28 | 107 | 135 | 21 |
| S10 | 58 | 75 | 133 | 44 |
| S11 | 22 | 57 | 79 | 28 |
| S12 | 27 | 176 | 203 | 13 |
| S13 | 24 | 38 | 62 | 39 |
| S14 | 18 | 58 | 76 | 24 |
| S15 | 22 | 17 | 39 | 56 |
| S16 | 26 | 164 | 190 | 14 |
| S17 | 53 | 155 | 208 | 25 |
| S18 | 16 | 54 | 70 | 23 |
| S19 | 18 | 22 | 40 | 45 |
| S20 | 19 | 76 | 95 | 20 |
| S21 | 19 | 71 | 90 | 21 |
| S22 | 21 | 45 | 66 | 32 |
| L1 | 19 | 199 | 218 | 9 |
| L2 | 47 | 61 | 108 | 44 |
| L3 | 60 | 122 | 182 | 33 |
| L4 | 14 | 27 | 41 | 34 |
| L5 | 22 | 96 | 118 | 19 |
| L6 | 67 | 72 | 139 | 48 |
| L7 | 97 | 135 | 232 | 42 |
| L9 | 70 | 214 | 284 | 25 |
| L10 | 27 | 223 | 250 | 11 |
| L11 | 74 | 85 | 159 | 47 |
| L13 | 37 | 197 | 234 | 16 |
| L14 | 34 | 263 | 297 | 11 |
| L15 | 32 | 163 | 195 | 16 |
| L16 | 57 | 95 | 152 | 38 |
| L17 | 74 | 116 | 190 | 39 |
| L18 | 31 | 159 | 190 | 16 |
| L19 | 50 | 162 | 212 | 24 |
| L20 | 55 | 116 | 171 | 32 |
| L21 | 16 | 127 | 143 | 11 |
| L22 | 27 | 172 | 199 | 14 |
| L23 | 31 | 165 | 196 | 16 |
| L24 | 37 | 143 | 180 | 21 |
| L25 | 33 | 133 | 166 | 20 |
| L27 | 25 | 126 | 151 | 17 |
| L28 | 20 | 121 | 141 | 14 |
| L29 | 30 | 145 | 175 | 17 |
| L30 | 25 | 89 | 114 | 22 |
| L31 | 19 | 64 | 83 | 23 |
| L31B | 14 | 85 | 99 | 14 |
| L32 | 18 | 108 | 126 | 14 |
| L33 | 12 | 55 | 67 | 18 |
| L34 | 8 | 24 | 32 | 25 |
| L35 | 10 | 7 | 17 | 59 |
| L36 | 13 | 55 | 68 | 19 |
| MBP | 122 | 61 | 183 | 67 |
| Trx | 129 | 216 | 345 | 37 |
| GST | 119 | 111 | 230 | 52 |
| NusA | 15 | 18 | 33 | 45 |
| Ub | 57 | 62 | 119 | 48 |
| DI-IF2 | 100 | 128 | 228 | 44 |
| EF-Tu | 95 | 177 | 272 | 35 |
| EF-P | 100 | 96 | 196 | 51 |
| IF1 | 51 | 97 | 148 | 34 |
| IF3 | 91 | 52 | 143 | 64 |
| NTL9 | 20 | 126 | 146 | 14 |
| ibpA | 18 | 207 | 225 | 8 |
| ibpB | 34 | 223 | 257 | 13 |
| skp | 58 | 111 | 169 | 34 |
| slyD | 165 | 117 | 282 | 59 |
| dsbA | 35 | 44 | 78 | 44 |
| dsbB | 39 | 253 | 292 | 13 |
| dsbC | 83 | 142 | 225 | 37 |
| secB | 56 | 94 | 150 | 37 |
| secE | 14 | 112 | 127 | 11 |
| secG | 17 | 98 | 115 | 15 |
| grpE | 90 | 78 | 168 | 54 |
| fkpB | 91 | 90 | 181 | 50 |
| fklB | 74 | 53 | 127 | 58 |
| groEL | 25 | 16 | 41 | 60 |
| groES | 53 | 156 | 209 | 25 |
| groEL191-345 | 18 | 19 | 37 | 49 |
| groEL191-376 | 11 | 11 | 22 | 51 |
| lysN | 46 | 33 | 79 | 58 |
| aspN | 91 | 185 | 276 | 33 |
| asnN | 21 | 36 | 57 | 38 |
