# Supplementary material for: Expression Screening of Fusion Partners from an E. coli Genome for Soluble Expression of Recombinant Proteins in a Cell-Free Protein Synthesis System
Source: PLoS One. 2011 Nov 2;6(11):e26875. doi: 10.1371/journal.pone.0026875 (PMC3206877; doi:10.1371/journal.pone.0026875)
Supplement: Table S5 — AGGRESCAN analysis. (DOC) [file pone.0026875.s006.doc]

**Table S5.** AGGRESCAN analysis.
